# The impact of inequality on social value orientation: an eye-tracking study

**DOI:** 10.3389/fpsyg.2025.1521101

**Published:** 2025-02-28

**Authors:** Qian-Hui Wang, Zi-Han Wei, Wan-Ning Chen, Yu Na, Hui-Ming Gou, Hong-Zhi Liu

**Affiliations:** ^1^Department of Social Psychology, School of Sociology, Nankai University, Tianjin, China; ^2^Key Research Base of Humanities and Social Sciences, Institute of Psychology and Behavior, Tianjin Normal University, Tianjin, China; ^3^College of Cryptology and Cyber Science, Nankai University, Tianjin, China; ^4^Laboratory of Behavioral Economics and Policy Simulation, Tianjin, China

**Keywords:** social value orientation, SVO ring, inequality, eye-tracking, information processing

## Abstract

**Introduction:**

Researchers have developed the social value orientation (SVO) framework to describe prosocial tendencies. However, existing tools for measuring SVO lack sufficient attention to the effect of option inequality, driven by the inequality-aversion motive. In this research, we conducted an eye-tracking experiment to compare the traditional SVO measure with the inequality-controlled condition, investigating how it influences estimated SVO values and underlying process mechanisms.

**Methods:**

A within-subjects eye-tracking experiment was conducted with 65 university students recruited from a university’s human subjects pool. Participants received 20 yuan (RMB; approximately US $2.9) in cash for their participation.

**Results:**

SVOs were lower in the inequality-controlled condition than in the traditional SVO measure. Information processing, including complexity, depth, and direction, differed when fairness was controlled. The predictive effect of relative time advantage was also enhanced under controlled inequality conditions. In addition, the predictive effect of relative time advantage was stronger when controlling for option inequality, suggesting that controlling for option inequality enhances bottom-up information processing.

**Discussion:**

These findings suggest that traditional SVO measures may overestimate prosocial tendencies due to a lack of inequality control. The study highlights the role of fairness evaluation in SVO assessments and provides insights into the cognitive mechanisms underlying prosocial decision-making, offering guidance for future SVO measurements.

## 1 Introduction

In complex social environments, individuals’ decisions can significantly affect not only their own welfare but also the well-being of others around them. Sometimes, individuals face choices where they might sacrifice their own resources to help others without expecting any direct benefits. Examples of such decisions include participating in a local blood drive, donating money to a stranger in need, or dedicating efforts to organize a community clean-up event. Classic economic theory traditionally assumes that people are purely selfish and focused on maximizing monetary gain, leading them to choose the option with the highest personal benefit while disregarding the impact on others. However, empirical research has consistently shown that many individuals consider the effects of their actions on others and exhibit prosocial behaviors (Bear and Rand, 2016; Camerer, 2011; Fehr and Fischbacher, 2003).

### 1.1 The social value orientation framework

Researchers have developed the framework of social value orientation (SVO) to describe the tendency of individuals to consider others’ welfare compared to their own (Liebrand and McClintock, 1988; Van Lange, 1999). SVO is viewed as a trait that reflects how individuals evaluate outcomes for both themselves and others (Messick and McClintock, 1968). Substantial research has demonstrated that SVO is a valid predictor of prosocial and cooperative behavior (Balliet et al., 2009). In addition, SVO has been found to affect cognition and account for interpersonal decisions such as resource dilemmas (Roch et al., 2000; Roch and Samuelson, 1997) and negotiation behaviors (De Dreu and Boles, 1998).

The SVO of an individual can be represented by the weights given to outcomes for oneself and another person. SVO is commonly measured using money allocation tasks, where participants decide how to distribute money between themselves and an anonymous participant (Liebrand and McClintock, 1988; Murphy et al., 2011). These tasks reveal individuals’ willingness to forgo their own money to increase other’s payment. According to the SVO framework, the utility derived from a monetary allocation is defined by the following function:


(1) $$U=w_{1}\times \left(\text{own}\;\text{payoff}\right)+w_{2}\times \left(\text{other}’s\;\text{payoff}\right)$$

where *w*_1_ and *w*_2_ indicate the decision weights for own and other’s payoffs, respectively. These weights allow for the calculation of a gradual measure of SVO, known as the SVO angle, using the function of arctan (*w*_2_ / *w*_1_). Participants’ SVO angles can be classified into four SVO types: altruists, cooperators, individualists, or competitors (Liebrand and McClintock, 1988; Murphy and Ackermann, 2014).

The earliest measure for SVO is the SVO Ring measure (Liebrand and McClintock, 1988). In this measure, individuals repeatedly select between two options that differ in payoffs for themselves and others. For example, an individual might choose between Option A: $100 for self and $50 for other, or Option B: $98 for self and $63 for other. In this situation, the individual could either maximize her own payoff by choosing Option A or sacrifice some of her money to increase the other person’s payoff by choosing Option B (Murphy and Ackermann, 2014). The SVO Ring measure consists of 24 decisions between two options, each with different outcomes. Participants’ SVO angles can be calculated based on the decision weights estimated by the Equation 1. Subsequent researchers have developed more advanced tools for measuring SVO, such as the Slider measure. Compared to other measurements, the Slider measure requires fewer items to measure individuals’ SVO and has higher test–retest reliability (Bakker and Dijkstra, 2021).

### 1.2 The insufficient attention to equality in SVO measurements

Some researchers argued that the utility for an allocation is determined not only by one’s own and other’s payoffs but also by *inequality aversion* (Fehr and Schmidt, 1999; Van Lange, 1999, 2008). This egalitarianism makes people to have the tendencies toward enhancing equality in outcomes for self and another person (Van Lange, 1999). Wei et al. (2023) suggested that the SVO framework may not sufficiently emphasize the role of equality, which is crucial in social decisions. For example, in the previously mentioned scenario, the inequality of Option A is $50 (i.e., $100 – $50 = $50), whereas the inequality of Option B is $35 (i.e., $98 – $63 = $35). An individual choosing Option B might do so not only out of altruism but also due to a preference for equality. This suggests that the inequality of the two options might confound participants’ altruistic tendencies, leading to inaccurate SVO measurements. To our knowledge, no studies have specifically evaluated the effect of equality on SVO measurements.

It is worth noting that in SVO measurement, a common distinction is made between different types of prosocial, such as cooperators, who aim to maximize joint outcomes, and altruists, who focus on maximizing the other’s payoff (Au and Kwong, 2004). These two types reflect different strategies for approaching situations of interdependence: cooperators prioritize collective payoffs (Bogaert et al., 2008; Van Lange et al., 2007), while altruists focus on the payoffs of others (Murphy and Ackermann, 2014). However, both strategies seem unable to capture the estimation of equality in the options, since both cooperator and altruist strategies focus more on the distribution of payoffs. In addition, besides altruists and cooperators, the competitive prosocial type deserves attention. Individuals with a competitive orientation aim to maximize the relative difference between their own and others’ payoffs, increasing their own while decreasing others’ (de Matos Fernandes et al., 2022; Murphy et al., 2011). Although this seems related to equality estimation, the correlation is weak. The reason is that, as the SVO formula shows, it does not estimate equality between options, but instead calculates SVO angles through the ratio. Therefore, existing SVO measurements lack sufficient attention to the estimation of equality.

In the present study, we aimed to investigate whether the inequality of the options influences participants’ estimated SVO values. In addition to using the SVO Ring measure, we conducted a series of pairs of allocations where the inequality between options was controlled. This approach allowed us to isolate the effect of option inequality. We then compared the estimated SVO values obtained from the SVO Ring (SVOR) measure and the inequality-controlled (IC) condition. Previous research has indicated that an aversion to inequality can drive social preferences (Fehr and Schmidt, 1999; Wei et al., 2023). Therefore, we hypothesized that the inequality of options would affect participants’ estimated SVOs. Our hypothesis is as follows:

*H1*: The SVOs estimated in the SVOR condition and the IC condition will differ.

### 1.3 Examining information processing in SVO measurement using eye-tracking technique

In recent years, researchers have employed eye-tracking technique to explore information processing during social decisions. For instance, Fiedler et al. (2013) recorded participants’ eye movements during the SVO Ring task, discovering that variations in SVOs corresponded with consistent differences in information search patterns. Similarly, Jiang et al. (2016) investigated eye movements in a three-person distribution experiment, finding that different social preference strategies—such as efficiency, maxi-min, and envy—were associated with distinct transition patterns. Wei et al. (2023) also found that the direction of information search correlated with choices in a mini-dictator game. These findings imply a potential link between information processing and social decisions. Therefore, it seems feasible to compare eye movements in the SVOR condition with those in the IC condition to provide further process evidence for the role of inequality aversion in the SVO measurement.

In the present research, we used eye-tracking technology to investigate the differences in cognitive processes between the SVOR condition and the IC condition. We hypothesized that the motive for inequality aversion plays a significant role in social preferences. Specifically, we predicted that the IC condition, where the inequality of options is controlled to be similar, would induce more decision conflicts compared to the SVOR condition, resulting in differences in eye-tracking measures between the two conditions.

Previous research has typically compared three aspects of information search and processing across different decision tasks: complexity level, depth, and direction (Liu et al., 2022; Su et al., 2013). In line with this, we focused on these three aspects to compare information processing between the two conditions. (1) The complexity level of information processing refers to the cognitive effort required to process and integrate information (Su et al., 2013). When decision criteria are similar among options, decision-making can become more challenging (Brandstätter et al., 2006), leading to higher complexity levels in information processing (Liu et al., 2022). This increased complexity can be observed through total fixation count, with more fixations indicating greater cognitive demand (Yang et al., 2022). We thus infer that compared to the SVOR condition, the IC condition, where the fairness among options is similar, will elicit greater complexity levels of information processing. (2) The depth of information acquisition pertains to the extent of information exploration (Su et al., 2013) and is measured by the amount of information searched upon before making a decision (Payne and Braunstein, 1978). It reflects how thoroughly participants explore and examine all available options and attributes before reaching a decision (Su et al., 2013). When the fairness among options is similar, participants are likely to search for more information to reach a decision. Consequently, we hypothesize that the depth of information acquisition will be greater in the IC condition compared to the SVOR condition. (3) The direction of information search is closely tied to decision strategies (Liu et al., 2021; Pachur et al., 2013; Zhou et al., 2022). Option-wise transitions correlate with evaluating inequality, while attribute-wise transitions correlate with evaluating one’s own and others’ payoffs (Wei et al., 2023). Previous research indicates that option-wise transitions may reflect assessments of fairness (Wei et al., 2023). When the fairness levels of options are similar, evaluating fairness becomes more challenging, leading to an increase in option-wise comparisons. Consequently, the similarity of fairness among options is expected to promote option-wise transitions. We thus hypothesize that participants in the IC condition will exhibit more option-wise information search compared to the SVOR condition. Based on these considerations, we propose the following hypotheses:

*H_2a_*: Participants will show a higher complexity level of information processing in the IC condition compared to the SVOR condition.

*H_2b_*: Participants will search for more information in the IC condition compared to the SVOR condition.

*H_2c_*: Participants will exhibit more option-wise transitions in the IC condition compared to the SVOR condition.

In addition, we will further examine the relationship between eye-tracking measures and social decisions at the trial level. We hypothesized that the two conditions would result in distinct eye-tracking measures, leading to different social decisions. Therefore, we propose the following hypothesis:

*H_3_*: The eye-tracking measures will mediate the effect of condition on social decisions.

### 1.4 Overview of the present study

In the present study, we conducted an eye-tracking experiment to compare the traditional SVO Ring measure with the inequality-controlled condition. Specifically, we investigated the differences in estimated SVO values (H_1_) and eye-tracking measures between the two conditions (H_2_) and tested the mediating effect of the eye-tracking measures (H_3_). Data from these two studies and supplemental materials are publicly available via the Open Science Framework.^1^

This study has three key innovations. First, it highlights that insufficient attention to equality in SVO measurements can conflate altruism with inequality aversion, thereby producing inaccurate estimates, and addresses this issue by introducing an inequality-controlled (IC) condition. Second, it directly compares the traditional SVO Ring measure with the IC approach, revealing how equality considerations influence social decision making. Third, it employs eye-tracking techniques to capture the complexity, depth, and direction of information processing, offering process-level evidence for the role of inequality aversion in shaping prosocial choices.

## 2 Method

### 2.1 Participants

We used G*Power software (Version 3.1.9.6; Faul et al., 2007) to calculate the sample size needed to achieve 0.95 power to detect a medium effect size (Cohen’s *d* = 0.5) using a paired *t*-test at a two-sided significance level of 0.05. The required sample size was *N* = 45. Therefore, a total of 65 college students (51% female, *M*_age_ = 21.8 ± 2.1) were recruited as participants from a university’s human subjects pool. Participants received 20 yuan (RMB; approximately US $2.9) in cash for their participation. All participants had normal or corrected-to-normal vision and provided written informed consent prior to the experiment. The study was approved by the university’s Institutional Review Board.

### 2.2 Apparatus

The stimuli were presented on a 17-inch LCD monitor with a resolution of 1,024 × 768 pixels and a refresh rate of 60 Hz, controlled by a Dell PC. During the trial, participants responded to the stimuli by pressing specific keys on the keyboard. Meanwhile, their eye movements were monitored by an EyeLink 1,000 Plus eye-tracker (SR Research, Ontario, Canada) with a sampling rate of 1,000 Hz. Participants’ eyes were approximately 60 cm from the screen, with a chin rest to minimize head movements. From this distance, the screen subtended a visual angle of 36° horizontally and 29° vertically. Since both eyes were fixed on the same area, eye movement data were collected only from the right eye. The experiment was controlled using SR Research Experiment Builder software (version 2.3.38).

### 2.3 Stimuli and task

In the experiment, we employed the binary money allocation paradigm, a common method to assess individual social preferences (Chen and Krajbich, 2018; Teoh et al., 2020; Wei et al., 2023). In each trial, participants were asked to make a series of choices between two options representing money allocations that differed in terms of payoffs to themselves and others. Participants were informed that both they and the other person would remain anonymous, and that the other person would not make choices for them. This method has been commonly used in previous research (Wei et al., 2023; Hu and Mai, 2021). Participants chose to either maximize their own payoff or sacrifice personal gain to benefit others. The center-to-center distance between any two payoffs exceeded 5° horizontally or vertically. This ensured proper fixation on payoff values and prevented peripheral identification of adjacent values during fixation (Rayner, 1998, 2009). The options for one’s payoff and the other’s payoff were vertically aligned, allowing placement on the left and right sides of the screen. In half of the trials, the participant’s payoffs appeared at the top for vertical alignment, while in the other half, the other’s payoffs were at the top.

The stimuli consisted of 88 pairs of options, which were divided into two conditions. The first condition, termed the SVO Ring (SVOR) condition, included 24 pairs of options.^2^ These options had been commonly used in previous research to evaluate participants’ social value orientations (Fiedler et al., 2013; Liebrand and McClintock, 1988). In the second condition, termed the Inequality Controlled (IC) condition, we constructed 64 pairs of options designed to control for fairness by ensuring that the absolute payoff differences between oneself and the other person remained equal across both options in each pair. Specifically, one option in each pair offered a higher payoff for oneself, while the other offered a higher payoff for the other person. The payoffs varied across trials to provide a diverse range of conditions while maintaining fairness, reducing potential bias from any single value combination. These pairs were generated using a computational method designed to fix the fairness within the pairs of options. The stimuli can be found in Supplementary Table S1.

To motivate participants, they were informed before the experiment began that two individuals would be randomly chosen to play the roles of divider and receiver. The outcomes of their choices in a randomly chosen trial would determine the additional rewards for both participants, directly linking their decisions to potential personal gains. To ensure credibility, the selected trial’s decision was actually implemented at the end of the experiment.

### 2.4 Procedure

Participants first signed an informed consent form and then received instructions about the experiment, including a brief description of the apparatus. Before the experiment began, participants were required to undergo an eye tracker calibration, involving a 5-point calibration and validation process. The calibration aimed to maintain the precision of measurements, with an acceptable maximum validation error set at 0.5° of visual angle. Calibration was recalibrated as necessary, particularly following a failed drift check. After calibration, participants read the instructions and engaged in two practice trials designed to acquaint them with the experimental task. The formal experiment comprised 88 trials, organized into two blocks of 44 trials each. The order of the trials was counterbalanced across participants. A two-minute break period was provided between the blocks to prevent fatigue.

**Figure 1:**
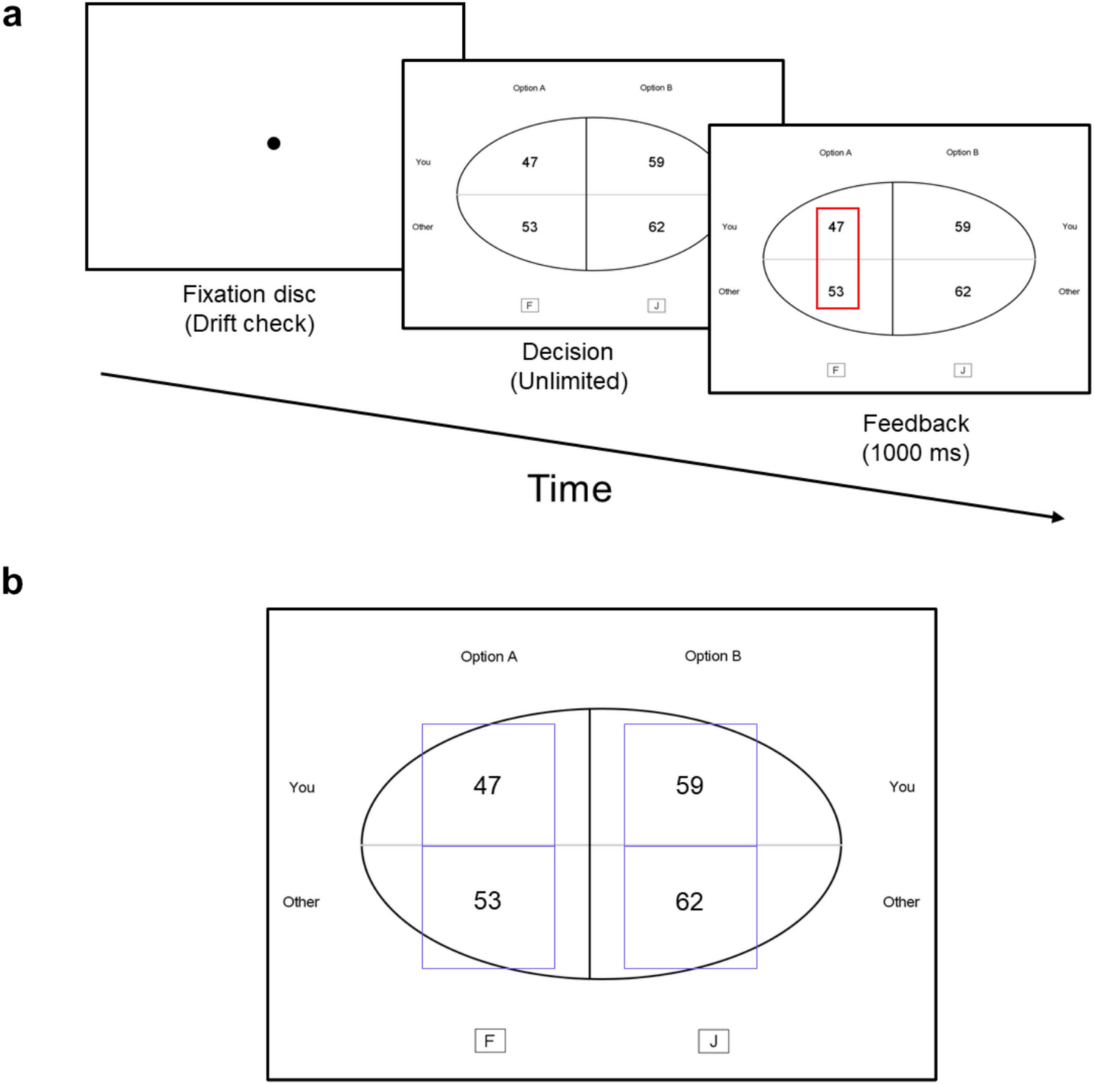
**(a)** Trial procedure and timing in the experiment. Each trial began with a fixation in the middle of the screen. After each response, a 1,000 ms feedback with a blank screen was presented before the next trial began. **(b)** Experimental stimuli. The blue rectangles around payoffs represent the regions of interest and were not visible to participants.

At the beginning of each trial, a circular fixation disc was displayed at the center of the screen for drift calibration. Participants triggered the presentation of stimuli by pressing the space key while fixating on the disc. Participants had no time constraints to choose between the two options, pressing “F” to choose the option on the left or “J” to choose the option on the right. Following each response, a feedback screen was displayed for 1,000 ms before the next trial began. Figure 1a depicts the trial procedure and timing.

### 2.5 Data analysis

#### 2.5.1 Estimating SVO

The SVO of an individual is quantified by the weights assigned to outcomes for oneself and for another individual, reflecting the degree of altruism or selfishness (Liebrand and McClintock, 1988; Van Lange, 1999). We used the following utility function to estimate participants’ SVOs:^3^


(2) $$U=\left(\text{own}\;\text{payoff}\right)+\alpha \times \left(\text{other}’s\;\text{payoff}\right)$$

Here, α represents the degree of altruism within the SVOs, gauging the intensity of altruistic behavior. Higher values of α indicate a stronger propensity towards altruism in the individual.

We adopted a hierarchical Bayesian approach to estimate SVOs from participants’ choice data across different conditions. This approach provides more reliable estimates of individual parameters by partially pooling them through group-level distributions, offering significant advantages over traditional, non-hierarchical methods (Nilsson et al., 2011).

To calculate the probabilities of choosing between options, we applied a *softmax* rule, which defines the probability that option A is chosen over option B as:


(3) $$p\left(A,B\right)=\frac{1}{1+e^{-\theta \left[U\left(A\right)-U\left(B\right)\right]}}$$

where *θ* (≥ 0) serves as a choice-sensitivity parameter. A higher θ increases the likelihood of selecting the option with greater utility, approaching certainty as increases; when θ = 0, choices are random.

For the estimation of joint posterior parameter distributions, we used Monte Carlo Markov Chain (MCMC) methods, implemented via JASP software (version 0.17.2.1). We ran three chains, each comprising 40,000 recorded samples, which were drawn from the posterior distributions after a burn-in period of 1,000 samples. The efficiency of these sampling procedures was validated through low autocorrelations among the sample chains and visual inspections of the chain plots.

#### 2.5.2 Preprocessing of eye-tracking data

Eye-movement data collected during the experiment were analyzed using DataViewer software (version 4.2.1). Fixations, defined as periods where gaze remained relatively stable between two saccades, were considered valid for analysis only if they lasted longer than 50 ms, with shorter fixations excluded to ensure data reliability. This is a typical method in previous research (Sui et al., 2020; Su et al., 2013). Four nonoverlapping rectangular regions of interest (ROIs), each measuring 6.7° × 6.7° in visual angle, were established around each piece of information presented (see Figure 1b). These ROIs were identically sized to maintain consistency in the analysis of gaze patterns across different types of information.

#### 2.5.3 Eye-tracking measures

Three eye-tracking measures were employed to test H_2_. First, the total fixation count (TFC) was defined as the total number of fixations recorded during a trial. The values of TFC are indicative of the complexity level of information processing (Yang et al., 2022). Previous studies have shown that TFC increases with task complexity (Malcolm and Henderson, 2010).

Second, the percentage of total information searched (PTIS) was used to gauge the depth of information acquisition in each task. In other words, PTIS quantifies the proportion of all predefined on which a participant fixates. For example, if four ROIs were defined in a given trial and a participant fixated on three of them, the PTIS would be 3/4 (0.75). PTIS is positively correlated with the thoroughness of information processing (Payne and Braunstein, 1978; Su et al., 2013). That is, a greater volume of information fixated upon before decision-making is associated with deeper information acquisition (Liu et al., 2022).

Third, the Payne Index (PI) was used to quantify whether transitions in gaze tend to be within options (e.g., from the own payoff of Option A to the other’s payoff of Option A, yielding a positive PI) or within attributes (e.g., from the own payoff of Option A to the own payoff of Option B, yielding a negative PI) (Amasino et al., 2019; Payne, 1976). The calculation of the PI was as follows:


(4) $$\text{PI}=\frac{\text{Option}\;\text{wise}-\text{transitions}-\text{Attribute}-\text{wise}\;\text{transitions}}{\text{Option}\;\text{wise}-\text{transitions}+\text{Attribute}-\text{wise}\;\text{transitions}}$$

#### 2.5.4 Data analyses

Data analysis was performed using *jamovi* (version 2.3.18) software (Şahin and Aybek, 2019). First, paired *t*-tests were conducted to examine differences between the two conditions. Additionally, Bayesian *t*-tests were utilized to evaluate the evidence supporting the alternative hypothesis. The default Cauchy prior width of 0.707 was used, and results were reported as *BF*_10_, which quantify the odds in favor of the alternative hypothesis over the null hypothesis based on the data. We categorized the strength of evidence using the terms “weak” (*BF* values from 1 to 3), “moderate” (*BF* values from 3 to 10), “strong” (*BF* values from 10 to 30), and “very strong” (*BF* values exceeding 30), following the classifications proposed by Jeffreys (1961).

Second, we investigated whether the effect of the experimental condition (SVOR vs. IC) on social decisions was mediated by eye-tracking measures. For this purpose, we employed the *GAMLj* module in *jamovi* software (Gallucci, 2019) to conduct a mediation analysis using a parallel multiple mediator model. This model not only allows for the correlation between mediators but also estimates the indirect effects of each mediator while controlling for the influence of others included in the model (Gallucci, 2019). We controlled for participant number (a discrete variable) and trial index (integers from 1 to 88) as covariates in our analysis. Confidence intervals of 95% were calculated based on 5,000 bootstrap samples.

Third, to examine the predictive effect of relative time advantage at the trial level, we used mixed effect models with the random effects of the participant number and trial index to analyze our data using *jamovi* software. Following the recommendations of Barr et al. (2013), the random effects structure was kept maximal for the models. As random effects, we included intercepts for both the participant and item and also by-participant random slopes for each fixed effect.

## 3 Results

Overall, 2 of the 5,720 trials were excluded from the analyses due to the eye-tracking failure, leaving a total of 5,718 valid trials.

### 3.1 Behavioral results

#### 3.1.1 Response time

Response times, defined as the total amount of time a participant took before making a decision, were log-transformed. Our analysis revealed no significant difference in response times between the SVOR condition (*M* = 2,203 ms, 95% confidence interval (CI) = [1958, 2,449] ms) and the IC condition (*M* = 2,273 ms, 95% CI = [2024, 2,521] ms), with results showing *t*_64_ = −0.29, *p* = 0.776, Cohen’s *d* = −0.04, *BF*_10_ = 0.14.

#### 3.1.2 The estimated SVOs

We used Equations 2, 3 to estimate the SVO parameters (*α*) and choice-sensitivity parameters (*θ*) for each participant. Analysis revealed no significant difference in the θ values between the SVOR condition (*M* = 13.7, 95% CI = [11.9, 15.6]) and the IC condition (*M* = 11.6, 95% CI = [7.9, 15.4]), *t*_64_ = 1.22, *p* = 0.227, Cohen’s *d* = 0.15, *BF*_10_ = 0.28. This suggested that choice sensitivities were similar across the two conditions.

**Figure 2:**
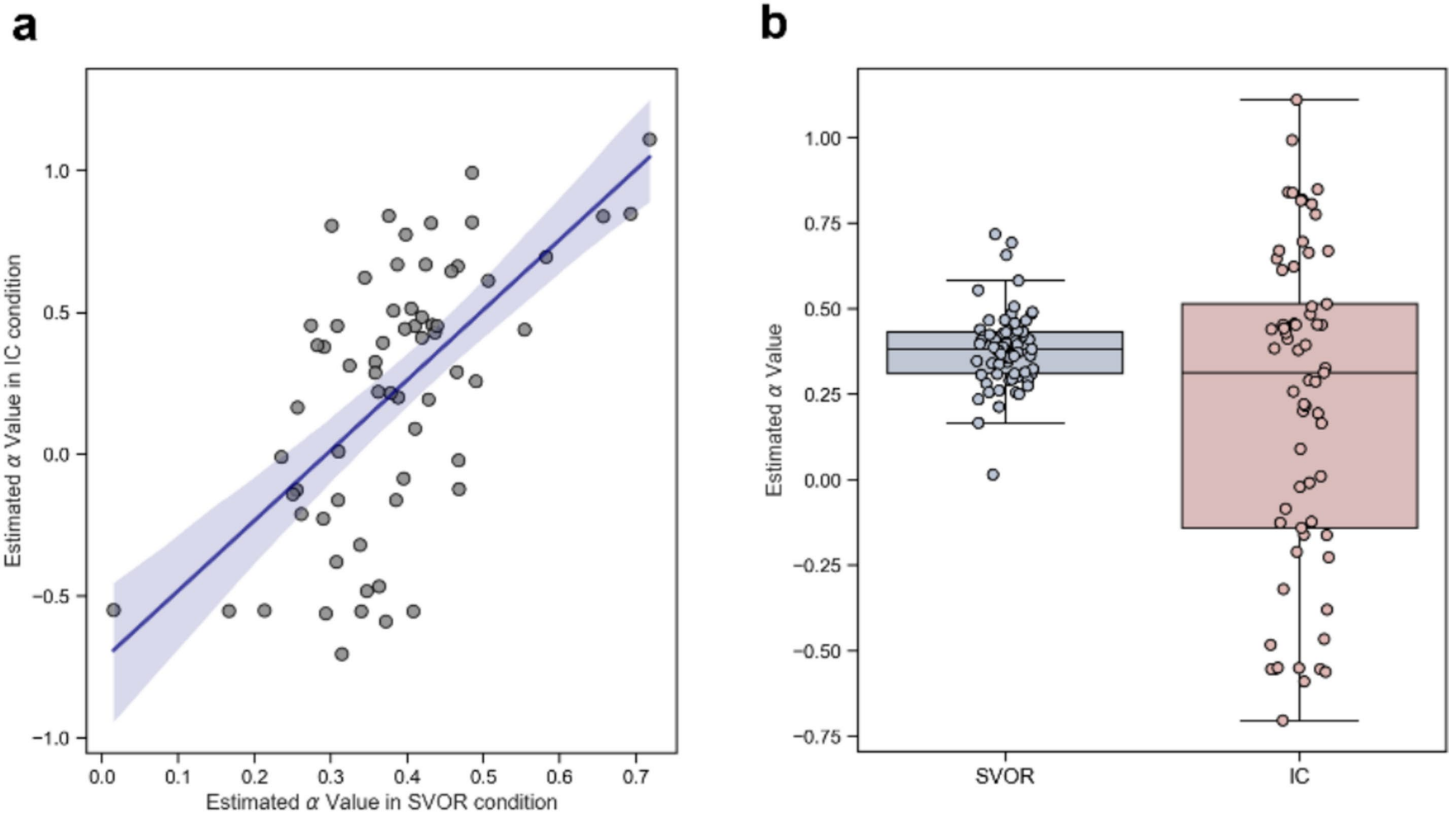
Results of estimated SVO parameters (*α* values) in the experiment. **(a)** Correlation between α values across the two conditions, with error bars representing 95%. **(b)** Comparison of α values showing higher values in the SVOR condition compared to the IC condition. The boxplot depicts the median as a center line, the quartiles as the bottom and top edges of the box, and the minimum and maximum values as whiskers.

Regarding the α parameters, our results indicated a significant correlation between the values estimated in the SVOR condition and those in the IC condition (Pearson’s *r* = 0.61, *p* < 0.001, see Figure 2a). However, α values were significantly higher in the SVOR condition (*M* = 0.38, 95% CI = [0.35, 0.41]) compared to the IC condition (*M* = 0.22, 95% CI = [0.10, 0.33]), *t*_64_ = 3.25, *p* < 0.001, Cohen’s *d* = 0.40, *BF*_10_ = 14.91, which indicates strong evidence for the alternative hypothesis as shown in Figure 2b.

### 3.2 Eye-tracking results

We compared three eye-tracking measures across the two conditions.

**Figure 3:**
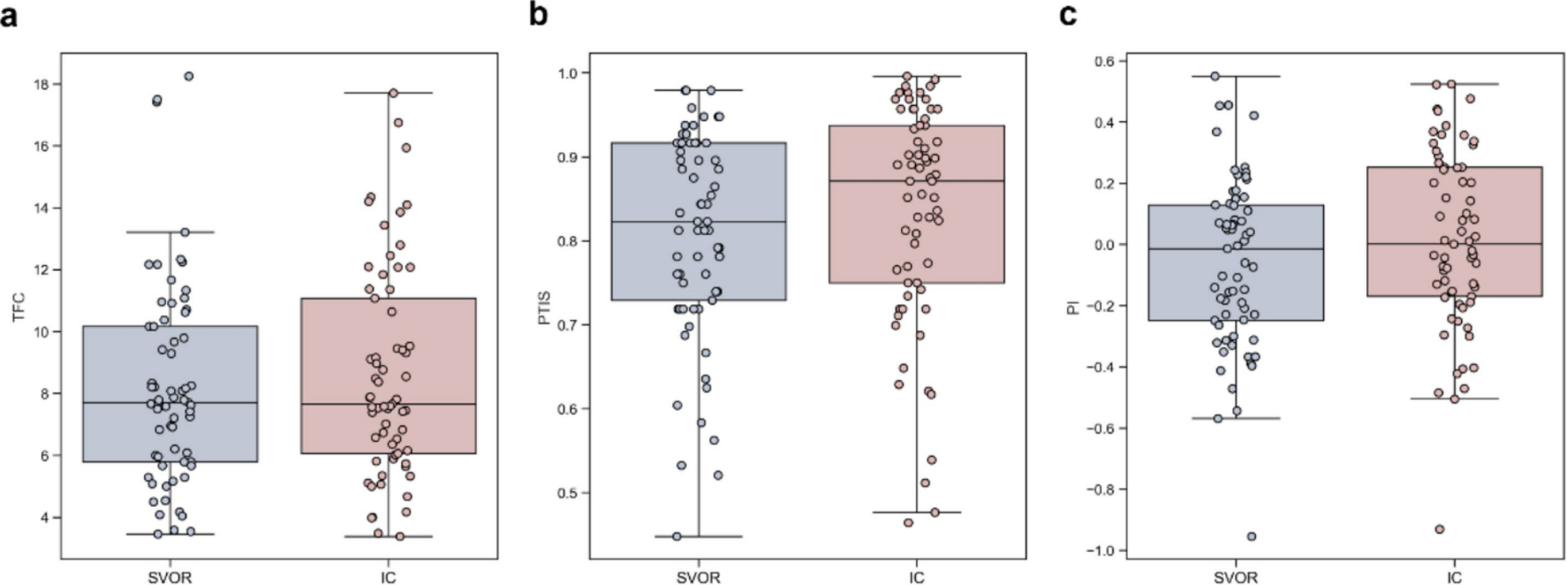
Results of eye-tracking measures in the experiment. The values of **(a)** total fixation count (TFC), **(b)** percentage of total information searched (PTIS) and **(c)** Payne Index (PI) were greater in the IC condition than in the SVOR condition. The boxplot depicts the median as a center line, the quartiles as the bottom and top edges of the box, and the minimum and maximum values as whiskers.

First, the TFC in the SVOR condition (*M* = 8.24, 95% CI = [7.43, 9.05]) was significantly lower than that in the IC condition (*M* = 8.61, 95% CI = [7.77, 9.44]), *t*_64_ = −2.15, *p* = 0.035, Cohen’s *d* = −0.27, *BF*_10_ = 1.16, which indicates weak evidence for the alternative hypothesis as shown in Figure 3a. This finding suggests that the complexity level of information processing in the SVOR condition was lower than that in the IC condition, supporting H_2a_.

Second, the PTIS in the SVOR condition (*M* = 80.7, 95% CI = [77.6, 83.7%]) was significantly lower than that in the IC condition (*M* = 83.2, 95% CI = [79.9, 86.5%]), *t*_64_ = −3.70, *p* < 0.001, Cohen’s *d* = −0.46, *BF*_10_ = 52.80, which indicates very strong evidence for the alternative hypothesis as shown in Figure 3b. This finding indicates that participants search for more information in the IC condition, and thus exhibited depth of information acquisition, compared to the SVOR condition, supporting H_2b_.

Third, the PI (as calculated in Equation 4) in the SVOR condition (*M* = −0.06, 95% CI = [−0.13, 0.01]) was significantly lower than that in the IC condition (*M* = 0.01, 95% CI = [−0.06, 0.09]), *t*_64_ = −4.26, *p* < 0.001, Cohen’s *d* = −0.53, *BF*_10_ = 301.04, which indicates very strong evidence for the alternative hypothesis as shown in Figure 3c. This finding suggests more option-wise search patterns in the IC condition than in the SVOR condition, supporting H_2c_.

### 3.3 Mediation analysis

To test whether the effect of the condition on choices was mediated by eye-tracking measures, we conducted a parallel multiple mediation model analysis. The condition (independent variable) was coded as a dummy-coded variable (SVOR = 1, IC = 0), and choices (dependent variable) were also coded as a dummy-coded variable (choose the selfish option = 1, choose the non-selfish option = 0). Consequently, logistic regression, rather than linear regression, was used for the analysis. The TFC, PTIS and PI were included as the mediators.

**Figure 4:**
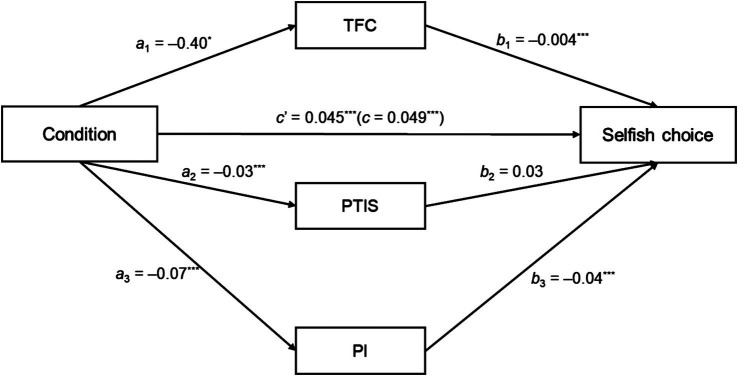
Results of the parallel multiple mediator model analysis of the **(a)** total fixation count (TFC), **(b)** percentage of total information searched (PTIS) and **(c)** Payne Index (PI). ^∗^*p* < 0.05. ^∗∗∗^*p* < 0.001.

The mediation analysis results are shown in Figure 4. The results revealed a significant indirect effect of condition on choices through the PI (*a*_3_*b*_3_ = 0.003, 95% CI = [0.001, 0.005], *z* = 2.71, *p* = 0.007). However, the indirect effects through the TFC (*a*_1_*b*_1_ = 0.002, 95% CI = [0.0002, 0.004], *z* = 1.85, *p* = 0.064) and the PTIS (*a*_2_*b*_2_ = −0.001, 95% CI = [−0.003, 0.001], *z* = −0.73, *p* = 0.465) were not significant. The total effect of condition on choices was *c* = 0.049, 95% CI = [0.022, 0.076], *z* = 3.59, *p* < 0.001. The direct effect of condition on choices, controlling for the mediators, remained significant (*c*’ = 0.045, 95% CI = [0.020, 0.071], *z* = 3.43, *p* < 0.001), indicating that it accounted for variance in choices beyond the effects of the mediators.

These results suggest that the PI mediated the effect of condition on choices. Compared to the SVOR condition, in the IC condition, the direction of information search was more option-wise, leading to an increase in selfish choices. Thus, H_3_ was partly supported.

### 3.4 The predictive effect of time advantage

**Figure 5:**
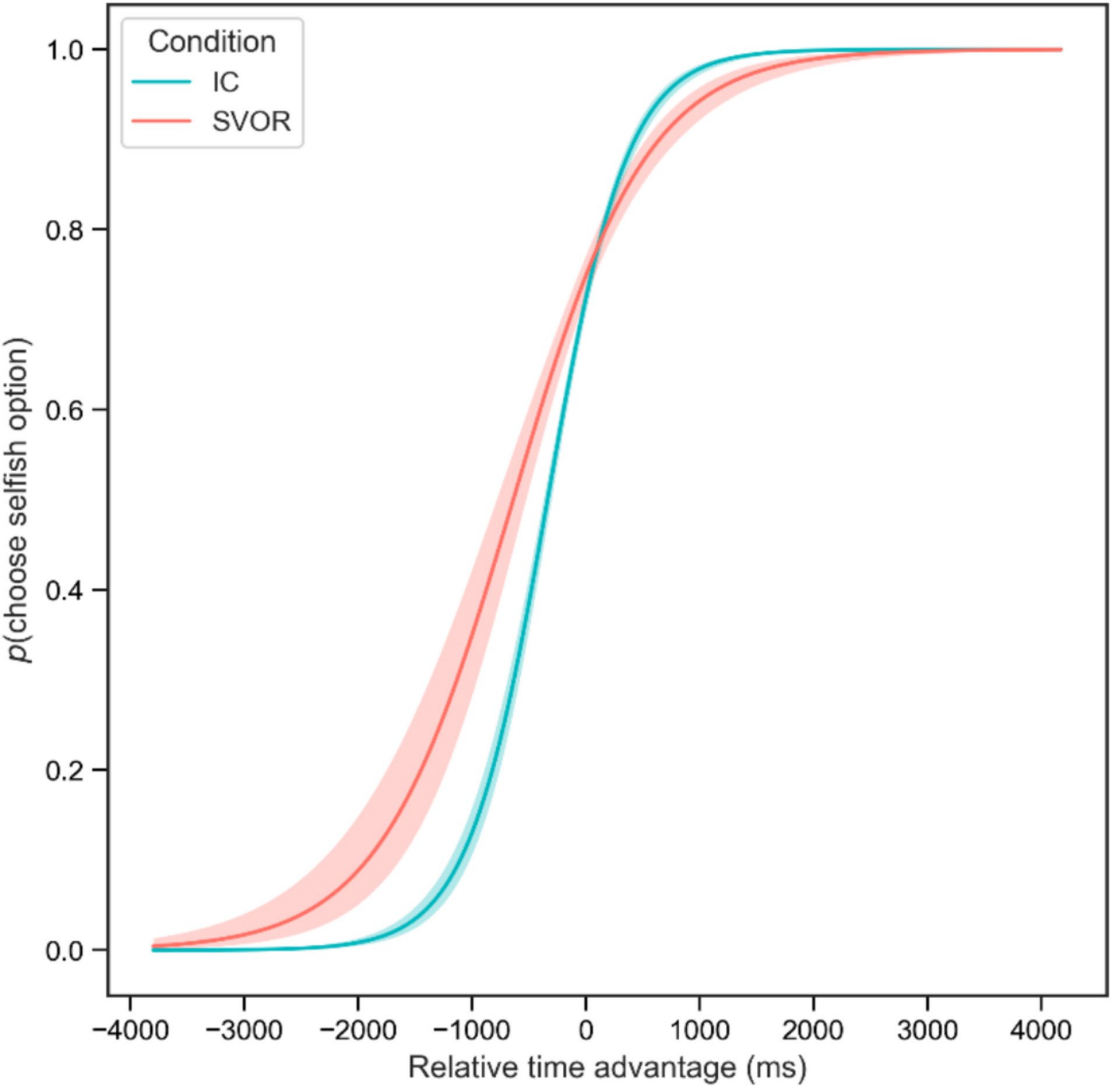
The predictive effect of time advantage on choices in the two conditions. Error bars represent 95% CI.

Characterized by an exploratory nature, we also attempted to test the predictive effect of time advantage in the two conditions. We conducted a mixed-effects logistic regression. The independent variables were the condition (dummy-coded, SVOR = 1, IC = 0), time advantage (the dwell time of the selfish option minus the dwell time of the non-selfish option, standardized), and their interaction. The dependent variable was participants’ choices (dummy-coded, choose the selfish option = 1, choose the non-selfish option = 0). The results revealed that the effect of condition was not significant (*b* = −0.16, exp.(*b*) = 0.85, 95% CI = [0.64, 1.14], *z* = −1.09, *p* = 0.275). However, the results revealed a significant effect of time advantage (*b* = 1.92, exp.(*b*) = 6.85, 95% CI = [4.95, 9.48], *z* = 11.59, *p* < 0.001) and a significant interaction (*b* = −0.49, exp.(*b*) = 0.61, 95% CI = [0.43, 0.87], *z* = −2.74, *p* = 0.006). Further simple slope analysis revealed that for the predictive effect of time advantage on selfish choices, the slope was stronger in the SVOR condition (*b* = 1.92, exp.(*b*) = 6.85, 95% CI = [4.95, 9.48], *z* = 11.59, *p* < 0.001) than that in the IC condition (*b* = 1.43, exp.(*b*) = 4.20, 95% CI = [2.90, 6.08], *z* = 7.57, *p* < 0.001). See Figure 5 for details.

## 4 Discussion

In this study, we conducted an eye-tracking experiment to investigate how the equality of options affects the estimation of SVOs. Our findings revealed that: (1) participants exhibited lower SVOs in the IC condition compared to in the SVOR condition; (2) there were significant differences in the complexity, depth, and direction of information processing when the fairness between options was controlled, and the direction of information processing mediated the effect of condition on choices; (3) the predictive impact of relative time advantage varied between the two conditions. These results highlight the critical role of option inequality in estimating SVOs.

### 4.1 Theoretical implications

The present study provides theoretical insights into understanding the mechanisms underlying SVO measurements in three aspects. First, this study identified the potential biases in traditional tools for measuring SVO, due to a lack of control over option inequality. Previous research has shown that inequality aversion drives social preferences (Fehr and Schmidt, 1999; Van Lange, 1999, 2008), which is consistent with our findings. In our study, we found that although the SVOs estimated under both conditions were correlated, the SVOs were significantly lower when the fairness of options was controlled. This indicates that while traditional SVO measures could capture individuals’ altruistic tendencies, these tendencies may be overestimated. For instance, an individual might be classified as “cooperative” based on her SVO angle (Liebrand and McClintock, 1988; Murphy and Ackermann, 2014). However, due to the overestimation of SVO, she might more accurately be classified as “competitive.” Given that numerous studies have investigated individuals’ SVOs using traditional tools, future research should reconsider the conclusions derived from these measures, particularly in light of potential confounds related to egalitarian motivation.

Second, our findings on eye-tracking measures provide further evidence on the information processing involved in eliciting SVOs. Specifically, our findings reveal that controlling for the fairness of options significantly impacts how individuals process information during allocation tasks. Participants exhibited greater complexity and depth in their information processing under the IC condition compared to the SVOR condition, suggesting that fairness control increases task difficulty. This observation aligns with previous research, which shows that higher complexity and depth of information processing are linked to increased task difficulty (Liu et al., 2022; Su et al., 2013). These findings indicate the role of inequality evaluation in allocation tasks and highlight the importance of understanding information processing strategies to explain variations in SVO, particularly in interdependent situations, thereby deepening our understanding of SVO beyond the simple classification of prosocial types. In addition, participants in the IC condition exhibited more option-wise transitions compared to the SVOR condition, and the direction of information search mediated the effect of condition on choices after controlling for other eye-tracking measures. This finding indicates that the direction of information search can predict participants’ social decisions at the trial level. Wei et al. (2023) demonstrated that the option-wise transitions correlate with inequality-aversion motivation. These findings suggest that in SVO measurement, different strategies lead to distinct information search methods, providing theoretical insight into the potential role of fairness in future SVO measurements and shedding light on the underlying process mechanisms.

Third, our findings indicate that the predictive effect of relative time advantage is stronger when controlling for the fairness of options. Although the relative time advantage predicted participants’ choices in both conditions, its effect was stronger in the IC condition than in the SVOR condition. Previous research has suggested that gaze-choice associations are often linked to decision quality (Sepulveda et al., 2020; Thomas et al., 2019). The reason for the stronger link between gaze and choice in the IC condition might be that participants invested more cognitive resources in their decisions in the IC condition. When individuals are more engaged in decisions, bottom-up information processing, such as visual factors of the stimuli, plays a more significant role in fostering decisions (Hu et al., 2023; Orquin et al., 2021). This is also confirmed by the fact that participants exhibited greater complexity and depth in information processing in the IC condition compared to the SVOR condition. It is thus inferred that measuring SVOs while controlling for the inequality of options may provide more accurate results.

### 4.2 Practical implications

This research presents practical implications for developing new tools to measure SVO. Existing tools, such as the SVO Ring Measure (Liebrand and McClintock, 1988), SVO Slider Measure (Murphy et al., 2011), and SVO Triple-Dominance Measure (Van Lange et al., 1997), do not control for the fairness of options, which may bias the measurement of participants’ altruistic tendencies. Although Van Lange (1999) pointed out that egalitarianism may be an important part of prosocial orientation, he did not propose a new measurement for SVO. Future studies developing new SVO measurement tools should control for the inequality of options, as in the IC condition in our experiment, to achieve more accurate measurements. Another approach is to incorporate inequality-aversion into SVO and construct a new three-dimensional framework, which may deepen the understanding of SVO.

### 4.3 Limitations

Finally, we acknowledge that this study has limitations. First, the two experimental conditions were not exactly matched, which may make the results in the two conditions less comparable. This is partly due to the inequality of options and the differences in own/other’s gains being covariant aligned, making it impossible to control for the inequality without altering these differences. Future studies may design more precise experiments to exclude potential confounding factors. Second, SVO as a stable personality trait (Murphy et al., 2011; Van Lange et al., 1997), exhibits substantial inter-individual heterogeneity (Fehr et al., 2002). Since the sample in this study was drawn from a university human subject pool, it may lack representativeness for the general population. Future research should aim to increase sample diversity to better reflect realistic situations.

## 5 Conclusion

In conclusion, our findings suggest that existing tools for measuring SVO may lead to biases due to a lack of control over the equality of options. Information processing, including complexity, depth, and direction, also varied when the fairness of options was controlled. This study provides insights into the potential role of fairness in future SVO measurements and sheds light on the underlying process mechanisms.

## Data Availability

The datasets presented in this study can be found in online repositories. The names of the repository/repositories and accession number(s) can be found in the article/Supplementary material.

## Footnotes

^1^ https://osf.io/2nheg/

^2^The reason for choosing the Ring measure over the Slider measure in this study is that the Slider measure requires participants to choose from nine options. This makes tracking more difficult due to excessive irrelevant data, which is not ideal for eye-tracking experiments.

^3^A common utility function used in the study of SVO is expressed in Equation 1, where the SVO angle can be derived from the weight parameters *w*_1_ and *w*_2_ (Liebrand and McClintock, 1988). However, following Krajbich’s (2021) recommendation, employing the *M* − 1 utility parameters (as per Equation 2) avoids the conflation of utility and consistency parameters, thereby reducing the risk of ambiguous interpretations and conclusions. Consequently, we utilized the *M* − 1 utility parameters function, which also allows for the calculation of the SVO angle based on the parameter *α*.
