# Supplementary material for: The impact of inequality on social value orientation: an eye-tracking study
Source: Front Psychol. 2025 Feb 28;16:1521101. doi: 10.3389/fpsyg.2025.1521101 (PMC11906464; doi:10.3389/fpsyg.2025.1521101)
Supplement: Supplementary file 1 [file Data_Sheet_1.PDF]

Supplemental Material for

The impact of inequality on social value orientation: An eye-tracking study

Table S1 Experimental stimuli

Item	Condition	A Own	A Other	B Own	B Other	A Equality	B Equality
1	SVOR	2	37	7	25	35	18
2	SVOR	85	15	93	25	70	68
3	SVOR	85	85	75	93	0	18
4	SVOR	15	15	25	7	0	18
5	SVOR	25	7	37	2	18	35
6	SVOR	2	63	0	50	61	50
7	SVOR	7	25	15	15	18	0
8	SVOR	63	2	75	7	61	68
9	SVOR	98	37	100	50	61	50
10	SVOR	15	85	7	75	70	68
11	SVOR	93	25	98	37	68	61
12	SVOR	7	75	2	63	68	61
13	SVOR	93	75	85	85	18	0
14	SVOR	50	100	37	98	50	61
15	SVOR	50	0	63	2	50	61
16	SVOR	0	50	2	37	50	35
17	SVOR	25	93	15	85	68	70
18	SVOR	37	98	25	93	61	68
19	SVOR	100	50	98	63	50	35
20	SVOR	37	2	50	0	35	50
21	SVOR	75	93	63	98	18	35
22	SVOR	63	98	50	100	35	50
23	SVOR	75	7	85	15	68	70
24	SVOR	98	63	93	75	35	18
25	IC	20	15	11	16	5	5
26	IC	24	15	16	25	9	9
27	IC	30	12	29	47	18	18
28	IC	33	28	32	37	5	5
29	IC	42	20	16	38	22	22
30	IC	45	15	36	66	30	30
31	IC	45	23	11	33	22	22
32	IC	46	16	20	50	30	30
33	IC	48	30	38	56	18	18
34	IC	50	16	33	67	34	34
35	IC	53	31	43	65	22	22
36	IC	54	16	37	75	38	38

37	IC	54	24	20	50	30	30
38	IC	54	32	36	58	22	22
39	IC	56	38	21	39	18	18
40	IC	58	36	57	79	22	22
41	IC	59	37	16	38	22	22
42	IC	59	41	42	60	18	18
43	IC	59	46	34	47	13	13
44	IC	60	26	18	52	34	34
45	IC	60	30	59	89	30	30
46	IC	64	30	29	63	34	34
47	IC	65	31	56	90	34	34
48	IC	66	40	65	91	26	26
49	IC	68	59	67	76	9	9
50	IC	69	14	10	65	55	55
51	IC	69	43	51	77	26	26
52	IC	70	23	11	58	47	47
53	IC	70	44	61	87	26	26
54	IC	71	41	53	83	30	30
55	IC	71	45	46	72	26	26
56	IC	72	34	46	84	38	38
57	IC	72	42	13	43	30	30
58	IC	73	35	14	52	38	38
59	IC	73	35	31	69	38	38
60	IC	73	35	39	77	38	38
61	IC	73	39	22	56	34	34
62	IC	73	64	56	65	9	9
63	IC	74	32	49	91	42	42
64	IC	76	29	33	80	47	47
65	IC	76	63	59	72	13	13
66	IC	78	36	28	70	42	42
67	IC	78	36	44	86	42	42
68	IC	79	49	37	67	30	30
69	IC	79	53	45	71	26	26
70	IC	80	25	29	84	55	55
71	IC	80	67	79	92	13	13
72	IC	81	30	38	89	51	51
73	IC	82	80	81	83	2	2
74	IC	83	49	57	91	34	34

75	IC	84	37	49	96	47	47
76	IC	84	50	25	59	34	34
77	IC	85	59	42	68	26	26
78	IC	85	72	76	89	13	13
79	IC	87	57	36	66	30	30
80	IC	89	30	29	88	59	59
81	IC	89	71	63	81	18	18
82	IC	92	50	33	75	42	42
83	IC	94	56	44	82	38	38
84	IC	95	44	43	94	51	51
85	IC	96	45	37	88	51	51
86	IC	97	71	46	72	26	26
87	IC	98	56	57	99	42	42
88	IC	99	52	48	95	47	47
